# Microbial Biodegradation of Synthetic Polyethylene and Polyurethane Polymers by Pedospheric Microbes: Towards Sustainable Environmental Management

**DOI:** 10.3390/polym17020169

**Published:** 2025-01-11

**Authors:** Maryam Najam, Sana Javaid, Shazia Iram, Kingkham Pasertsakoun, Marianna Oláh, András Székács, László Aleksza

**Affiliations:** 1 Department of Environmental Sciences, Fatima Jinnah Women University, The Mall, Rawalpindi 46000, Pakistan; maryam.najam@fjwu.edu.pk (M.N.); sana.javaid@fjwu.edu.pk (S.J.); 2 Institute of Environmental Sciences, Hungarian University of Agriculture and Life Sciences, Páter Károly u. 1, H-2100 Gödöllő, Hungary; kingkham.pasertsakoun@phd.uni-mate.hu (K.P.); olah.marianna@uni-mate.hu (M.O.); szekacs.andras@uni-mate.hu (A.S.); 3 Profikomp Environmental Technologies Inc., Kühne Ede u. 7, H-2100 Gödöllő, Hungary

**Keywords:** polyurethane, polyethylene, biodegradation, microbial degradation, soil microbes, sustainability

## Abstract

This study attempted to isolate and identify pedospheric microbes originating in dumpsites and utilized them for the degradation of selected synthetic polymers for the first time in a cost-effective, ecologically favorable and sustainable manner. Specifically, low-density polyethylene (LDPE) and polyurethane (PUR) were converted by the isolated fungi, i.e., *Aspergillus flavus*, *A terreus*, *A. clavatus*, *A. nigers* and bacterial coccus and filamentous microbes and assessed in a biotransformative assay under simulated conditions. Commendable biodegradative potentials were exhibited by the isolated microbes against polymers that were analyzed over a span of 30 days. Among the selected fungal microbes, the highest activity was achieved by *A. niger*, expressing 55% and 40% conversion of LDPE and PUR, respectively. In the case of bacterial strains, 50% and 40% conversion of LDPE and PUR degradation was achieved by coccus. Fourier transform infrared spectroscopy (FT-IR) and thermogravimetric analysis (TGA) were utilized to analyze the degradative patterns in terms of vibrational and thermal characteristics, and stereomicroscopic analysis was performed for the visual assessment of morphological variations. Profound structural transformations were detected in FT-IR spectra and TGA thermograms for the selected microbes. Stereomicroscopic analysis was also indicative of the remarkable transformation of the surface morphology of these polymers after degradation by microbes in comparison to the reference samples not treated with any pedospheric microbes. The results are supportive of the utilization of the selected pedospheric microbes as environmental remediators for the cleanup of persistent polymeric toxins. This current work can be further extended for the successful optimization of further augmented percentages by using other pedospheric microbes for the successful adoption of these biotechnological tools at a practical level.

## 1. Introduction

Environmental degradation in the current era can be attributed to a number of anthropogenic activities that give rise to either permanent or long-term changes in the ecospheric regime. Such practices are not only harmful towards human beings and other life forms, but, in an uncontrolled manner, they are destructive to the planet’s ecological integrity [1,2]. This includes the utilization of synthetic products in the form of daily use commodities, such as plastic bags, which are known for their persistent or potentially persistent nature [3]. The issue of plastic waste due to human activity is widespread throughout the environment. In 2019 alone, 460 million tons of plastic was produced, and 353 million tons were discarded in a single year. This amount is expected to triple by 2060 [4]. Great efforts have been devoted to the fulfillment of human needs in terms of rigorous research that has been conducted on food and energy [5,6,7,8], in addition to the environmental detoxification of different pollutants [6,8,9,10]. Some of these pollutants require urgent attention due to their toxic and persistent nature, in addition to the constant, numerous forms of damage they cause to humans and other life forms. Polymeric pollution due to plastics is a significant issue faced by humanity in the present era [11]. Life without any aspect of utilization of polymeric substances seems to be unattainable due to the complete domination of those products in a multiplicity of forms [12].

Polymers are formed from monomeric units linked in a repetitive manner, either in a linear or branched morphology [13,14]. The effectiveness and large-scale utilization of polymers can be attributed to their ease of fabrication, stability, flexibility, durability and other favorable physical properties [15,16]. Plastics and relevant products have become an important part of human life. However, the type of pollution associated with such products cannot be ignored. They can enter lithospheric and hydrospheric zones in the form of plastic debris from a wide range of products, such as ropes, plastic bags, fishing nets or other household objects or occupational equipment, and remain there for periods of unprecedented durations. Fifty percent of plastic materials are considered disposable, including utensils, packaging and plastic bags [17]. However, such disposability does not mean the complete dissipation of polymeric waste. The rate of degradation of polymeric waste is dependent upon a number of factors, including the environmental, climatic and microbial characteristics of the region in which the waste is deposited [18,19,20,21]. Thus, the accumulation of plastic waste is one of the most pressing environmental challenges globally, affecting ecosystems and economies alike [22].

In the utilization of plastic waste, material recycling takes priority, followed by chemical recycling (pyrolysis) and biodegradation [23,24]. Biodegradation processes can be triggered by a variety of fungal and bacterial cultures, depending on the physicochemical characteristics of the plastic materials to be degraded. They also depend on environmental and physical factors, such as pH, temperature, the availability of nutrients, inducers, inhibitors, the source of carbon, the presence of the contaminant, etc. [25,26,27,28,29]. The molecular weight of the polymer is another decisive factor for the determination of the fate of polymers in the environment; i.e., polymers with a high molecular weight are less susceptible to degradability than those with a low molecular weight [30]. Temperature is a crucial parameter affecting biodegradation rates, as most studies indicate that microbial degradation takes place at a temperature of 20 °C or higher, while a recent study identified 34 cold-adapted microbial strains when examining the plastisphere of polymeric materials buried in alpine and arctic soils [31]. Biodegradability was found to be inversely proportional to molecular weight in persistent and biodegradable homopolymers in different organisms in both terrestrial and aquatic ecosystems [32,33,34,35,36]. Furthermore, the degradation mechanism is also dependent upon the type of organism being used for polymeric waste degradation.

The main obstacles that render the biotechnological recycling of plastic waste unsustainable are low efficiency and slow degradation. Consequently, an intensive search for microorganisms—often originating from environmental sources or insect digestive tracts—capable of decomposing polyethylene, polypropylene, polystyrene, polyethylene terephthalate, polyvinyl chloride, polytetrafluoroethylene, and polyurethanes (PURs) is being undertaken [37,38,39,40]. However, some opinions suggest that the real utility of microbial decomposition is overemphasized [41]. PURs, a versatile group of polymers, significantly contribute to this issue due to their widespread use and resistance to degradation. Representing 7.7% of global plastic production, PURs are particularly resistant to biodegradation due to their varied chemical structures, composed of various isocyanates and polyols [42]. Recent studies, however, have made strides in investigating the biodegradation of PURs by microorganisms, fungi, and enzymes, offering promising insights into sustainable waste management solutions [43,44,45,46,47,48,49,50]. The susceptibility of plastics to degradation largely depends on their chemical structure. Nonetheless, some bacteria and fungi have demonstrated the ability to break down PURs under specific conditions. Several research studies have focused on the potential of microbial consortia to degrade synthetic polymers for use in bio-recycling processes [51]. Fungi, particularly from the *Ascomycota* phylum, have shown significant potential in degrading PURs. Fungi such as *Aspergillus, Cladosporium*, and *Penicillium* secrete specific enzymes such as hydrolases and ureases that contribute to the breakdown of plastic polymers [52,53]. The formation of biofilms by these fungi facilitates the degradation of PURs through the penetration of their enzymes into the material’s structure. Soil microorganisms were found to be capable of mineralizing aliphatic PUR foams at a rate of 30–50% over six months, indicating that fungi and bacteria in soil environments can utilize PURs as both an energy and nutrient source, primarily carbon [25,26,28,29]. These findings underscore the significance of soil microorganisms in the natural degradation of synthetic polymers [54].

While significant progress has been made in understanding the microbial and enzymatic degradation of PURs, challenges remain. Microbial degradation is slower compared to physical and chemical methods; however, it holds great promise for long-term sustainability. Several types of enzymes are known to be involved in the degradation of polyurethanes, all of them belonging to the hydrolase (EC 3) enzyme class. Since polyurethanes are polymers containing carbamate links among their monomers, both ester hydrolases (EC 3.1) and non-peptide amidases (EC 3.5) can degrade them. Among these, the two most important types are carboxylic ester hydrolases (EC 3.1.1) and urea amidohydrolases (EC 3.5.1). Within the group of carboxylic ester hydrolases, carboxylesterases (EC 3.1.1.1) and lipases (EC 3.1.1.3) play significant roles. Interestingly, cutinases (EC 3.1.1.74) have increasingly been reported to hydrolyze polyurethanes. Enzymes termed polyesterases and polyurethanases degrade polyurethanes but are not classified within the enzyme nomenclature database [55,56]. Polyurethanases, such as the enzyme produced by *Pseudomonas chlororaphis*, directly degrade polyurethane molecules [22,29,51,52,53,57,58]. Polyesterases, like those produced by *Thermobifida fusca*, are specialized for hydrolyzing polyesters but can also degrade certain forms of polyurethanes [59,60]. Esterases, including enzymes produced by *Thermobifida alba*, and lipases, such as *Candida antarctica* lipase B, primarily contribute to the degradation of polyester-based polyurethanes [48,53,57,60]. Ureases, like those produced by *Bacillus megaterium*, can also break down the urethane moiety [51]. Cutinases, such as those produced by *Thermobifida cellulosilytica*, exhibit broader substrate specificity and are capable of degrading both polyesters and polyurethanes [61,62,63,64]. Enzymatic degradation of PURs has gained attention as a potential method for reducing plastic waste. An enzymatic pre-treatment strategy has been proposed, utilizing enzymes to promote surface oxidation and improve the hydrophilicity of plastics, thereby enhancing microbial attachment and accelerating biodegradation [22,27,65,66]. This approach offers an environmentally more friendly alternative to traditional chemical and physical treatments, which often result in secondary pollution and high energy consumption. Furthermore, enzymes such as polyester-degrading esterases and urethane bond-degrading amidases have been shown to play crucial roles in PUR degradation in *Streptomyces* species [58,67]. Proteomic analyses revealed that PUR degradation intermediates are incorporated into bacterial metabolic pathways, offering insights into how these microorganisms can be leveraged for industrial-scale plastic recycling [68]. The most effective solutions can be achieved by integrating various techniques. Combining microbial degradation with enzymatic pre-treatment or physical techniques can accelerate the process and enhance the overall efficiency of PUR waste treatment [21,52,57,69,70].

The pedospheric zone, which is a recipient of a number of pollutants and toxins, contains entities that can be effectively utilized for environmental detoxification [6,7,71,72,73,74,75,76]. The soil profile serves as a breeding ground for the growth and sustenance of various microorganisms with diverse metabolic pathways. These microbes can serve as biotechnological tools capable of degrading and transforming different types of toxins, including polymeric materials, by releasing enzymes that convert intact substances into byproducts [58,65,77,78,79]. The consumption of polymers by pedospheric microbes to meet their energy needs leads to structural variations in the polymers, such as reduction in molecular weight. These structural and morphological changes can be observed using ultraviolet spectroscopy or other standard testing methods. The initiation of polymer degradation is marked by the effective conversion of a large macromolecular unit into smaller repetitive units through the process of mineralization [25,26,29]. Microbe-driven transformation mechanisms for various contaminants, including polymeric degradation, can be divided into four general steps: adherence, assimilation, fragmentation and mineralization [43,80,81,82].

Currently, reported cases of environmental deterioration due to plastic pollution present a significant challenge for humanity, particularly given the remarkable persistence of polymers in the ecosystem [17]. Human responses to such environmental deterioration should prioritize completely avoiding the use of toxic products, including polymeric substances, and focus on identifying suitable green alternatives for plastics [27,83,84,85]. However, considering the widespread use of plastic commodities and their affordability, developing viable alternatives requires extensive research and capacity building. Under these circumstances, where plastic pollution has become an unavoidable issue, human efforts should center on the adoption of greener technologies for the prevention and minimization of plastic pollution. To date, numerous microbial species have been utilized for the degradation of polymeric substances [86,87].

The objective of this study was to isolate and identify polymer-degrading fungi and bacteria from a dumpsite in Rawalpindi and to develop a cost-effective framework for the microbial degradation of polymers. Additionally, the efficiency of fungal and bacterial species in degrading polymers was quantified through analysis. This research paves the way for further studies in this field, particularly in developing countries like Pakistan, where waste management is usually neglected.

## 2. Materials and Methods

### 2.1. Materials

The synthetic polymers selected for current investigation were low-density polyethylene (LDPE), represented by plastic shopping bags, and PUR, represented by plastic foam. A local dumpsite located in the vicinity of Rawalpindi, Pakistan (33.5651° N, 73.0169° E), was selected as a testing site for the isolation of fungal and bacterial cultures. The isolated microbial cultures were identified using both microscopic and macroscopic methods to examine a wide range of morphological and structural features. The microbial species derivation process involved several steps, including isolation, culturing, the identification of species, sample preparation, the addition of samples to a salt solution culture medium (a modified Czapek Dox liquid medium containing 2.0% NaNO_3_, 0.7% KH_2_PO_4_, 0.3% K_2_HPO_4_, 0.5% KCl, 0.5% MgSO_4_.7H_2_O, 0.1% FeSO_4_.7H_2_O and 0.05% NaN_3_), inoculation of the salt solution and, finally, incubation of the sample for one month. This process was followed by various analyses, including weight loss measurements, Fourier transform infrared spectroscopy (FT-IR), thermogravimetric analysis (TGA) and stereo and electron microscopic analysis. All of the chemicals used in this current research were of analytical grade, with 99.99% purity, and were utilized without further purification. Deionized water (DW) was used for all the experiments.

### 2.2. Pedospheric Sampling

**Figure 1:**
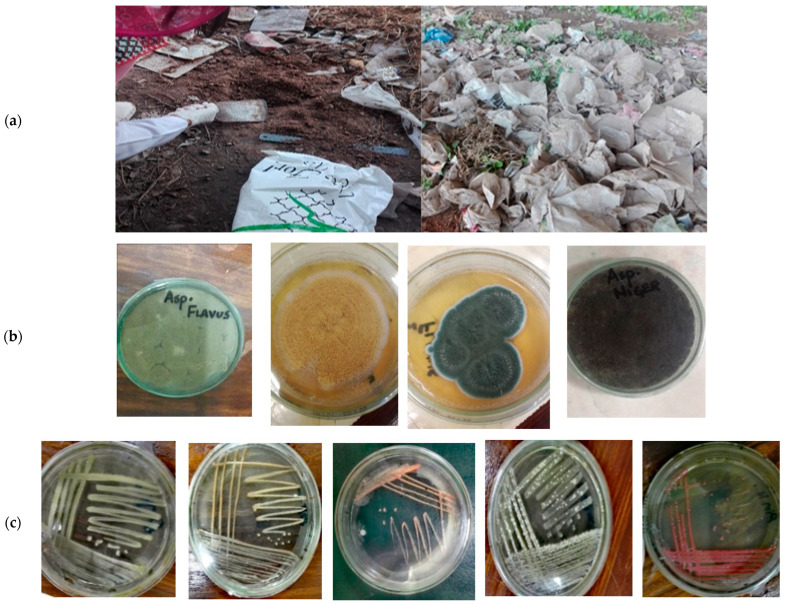
Pedospheric microbial sampling, isolation and identification: (**a**) sampling site at a dumping place at Rawalpindi, Pakistan, showing plastic garbage; (**b**) fungal strains, i.e., *Aspergillus flavus*, *A. terreus*, *A. clavatus* and *A. niger*; and (**c**) bacterial strains, i.e., white, light-yellow, orange, pink and dark-yellow bacterial colonies.

A total of three sampling sites were selected for soil collection in Rawalpindi, Pakistan (Figure 1). Soil samples were collected at a depth of 10 cm, placed in zip-lock bags and transported to the laboratory for further analysis. After transportation, the soil samples from the selected dump sites were mixed in equal proportions to prepare a composite sample, which was then sieved. After the sieving process, three different soil dilutions were prepared by adding 1 g of soil from each site to 100 mL of DW for fungal and bacterial cultures coded as F1, F2 and F3 for fungal cultures and B1, B2 and B3 for bacterial cultures. For fungal cultures, the standard spread plate method was applied, while for bacterial cultures, the standard pour plate method was used.

### 2.3. Microbial Strain Selection and Micro/Macro Identification

The microbial cultures selected for polymer degradation included both fungal and bacterial cultures. All of the experiments were performed in triplicate, and average values were calculated. The initial fungal plates displayed colonies with varying growth patterns. A small sample from each colony was collected using a sterilized needle and inoculated onto freshly prepared plates containing isolates of the pure fungal colonies on potato dextrose agar (PDA). A total of four distinct fungal colonies were isolated, identified and used for polymeric degradation of LDPE and PUR, as shown in Figure 1. Fungal strains were identified using the microtiter plate procedure of Dobranic and Zak [88], employing the Biolog Microbial Identification System (Biolog, Inc., Hayward, CA, USA). This system examines the ability of the microbes to assimilate or oxidize substances from pre-selected panel carbon sources. The Biolog Microplate tests generated characteristic wells with turbidity changes, leading to the formation of a metabolic fingerprint. The panel, comprising 95 types of biochemical tests, facilitated fungal strain identification through a standardized micro-method. PU and LDPE degradation was analyzed using weight loss measurements, FT-IR using an FTIR-8400 spectrometer (Shimadzu Corp., Kyoto, Japan), stereomicroscopic analysis and TGA using a TGA 8000 thermogravimetric analyzer (PerkinElmer Inc., Shelton, CT, USA) based on methodology from the scientific literature [89]. Additionally, selected polymer samples were further examined using scanning electron microscopy (SEM) with a BK-EM6900 (KYKY Technology Co., Beijing, China) scanning electron microscope operated at 20 kV.

Bacterial isolates that exhibited clear zones of hydrolysis around their colonies, indicating PU degradation, were selected for further investigation. These isolates were identified through macroscopic characteristics (colony morphology, pigment, shape, size, margin, surface), microscopic features (Gram staining, shape, cell arrangement, granulation, spore presence, motility) and biochemical properties, following the standard determinative bacteriology guidelines [90]. Correct identification of the fungal strains in the current work was accomplished based on the scientific literature [88,89,91], via reading and comparison between the phenotypic fingerprint of each plate and the Biolog Microbial Identification Systems Database. Consequently, the identified fungal cultures were *Aspergillus flavus*, *A. terreus*, *A. clavatus* and *A. niger*.

In addition to fungal cultures, various bacterial colonies were obtained through the pour plate method using nutrient agar (NA). Five distinct bacterial colonies appeared on the Petri dishes, which were part of the triplicate sets. All the colonies exhibited considerable differentiation, as reflected in their respective morphological traits. A single isolate of each bacterial colony was collected using a sterile loop and streaked onto freshly prepared nutrient agar plates [89]. The five bacterial cultures used for polymeric degradation are shown in Table 1 and Figure 1.

Macro-level identification of cultures was carried out based on their morphological characteristics, such as color, shape and size, as observed with the human naked eye, following methods in the scientific literature [88,89,91]. For instance, observations included the color and shape of fungal colonies, etc. Bacterial colonies were also distinctly visible to the naked eye [91]. Micro-level identification included characterization with the help of a light microscope.

### 2.4. Inoculum Preparation

The inoculum density was standardized for both fungal and bacterial cultures to ensure uniform exposure to the polymeric materials (LDPE and PUR). Fungal and bacterial inoculums were prepared in PDA and NA, respectively. Each inoculum was prepared in conical flasks by adding a small cultured sample of the respective fungal or bacterial culture using a sterilized needle, into 100 mL corresponding broths. PDA broth inoculums were placed in a shaker at 120 rpm and 35 °C for 72 h [89]. Selected polymeric samples, LDPE and PUR, were then aseptically added to the solution [92]. For the experiment, 24 conical flasks for fungal cultures and 30 conical flasks for bacterial cultures were prepared, each containing 50 mL of the solution. Each flask was loaded with one sample of PUR and one sample of LDPE. These flasks were placed in a shaker at 120 rpm and 35 °C temperature for 48 h. After two days, the treated samples were removed from the shaker and analyzed. Inoculum homogeneity was ensured by thoroughly mixing the inoculated broth before distributing it into the conical flasks. Additionally, microbial growth in the broth was periodically assessed by sampling and confirming uniformity in colony-forming unit counts (in the range of 2–8 × 10^9^ cfu/mL) through plating on nutrient agar. Optical density measurements at a wavelength of 600 nm were also conducted to verify consistent inoculum density across all flasks. This ensured uniform microbial exposure to the polymeric materials during the degradation process. The degradation of polymeric samples, LDPE and PUR, was studied using the salt solution listed in Table 1.

### 2.5. Sample Analysis Harvesting

After the incubation period, the growth of each species was clearly observed in each media bottle. Pre-weighed LDPE and PUR samples were added to 50 mL of a mineral salt solution, followed by the addition of 10 mL of inoculum into the conical flasks. These flasks, prepared for each species, were analyzed over the course of one month. Each flask was labeled with the specific fungal or bacterial species, corresponding to weekly sample intervals. These flasks were incubated for one month at 120 rpm and 35 °C. After the incubation period, the samples were harvested. The collected samples were washed first with distilled water and then with 70% ethanol before being dried under laminar airflow for 24 h [93].

### 2.6. Biodegradative Potential

To accurately estimate the activity of the selected pedospheric microbial cultures against LDPE and PUR, various analytical tools and measurements were employed. The weight loss of polymer was measured by first determining their initial weights before any microbial action on the samples. After one month of incubation, the dried samples were weighed again to assess the degradation rate using a comparison method [94]. The weight loss of selected polymers was calculated using Equation (1) [95].

(1) $$Weight\;loss=\frac{W_{1}-W_{2}}{W_{1}}x100$$

where W_1_ is the initial weight, and W_2_ is the final weight of the polymer.

After the four-week incubation of PUR and LDPE films in a liquid medium, these samples were then subjected to the FT-IR to evaluate degradation based on the variations in the functional group through vibrational analysis. TGA was also conducted to perform thermal analysis of microbe-driven polymeric degradation. This method monitored the physicochemical properties of the materials as a function of rising temperature (continuous heating rate) or time (continuous mass loss). The temperature range for the experiment was 25 °C to 600 °C for LPDE and PUR under an inert N_2_ atmosphere. A stereomicroscope was used to study the surface of the solid specimens. Prepared samples were placed under a microscope to examine the surficial parameters, highlighting the occurrence and extent of biodegradation after microbial treatment.

## 3. Results and Discussion

Biodegradation is an eco-friendly, sustainable and cost-effective approach to plastic pollution prevention and minimization. This current work effectively utilized microbial biotechnological tools derived from the pedospheric zone of a dumpsite for the degradation of polymeric pollutants. To assess PUR and LDPE biodegradation by microbial strains, different testing methods, including weight loss measurements, FT-IR, TGA and stereo and electron microscopic analysis were employed [41]. The fungus *Aspergillus versicolor* isolated from soil was found to degrade up to 55% of PURs after one month of exposure [21]. Similarly, *Tenebrio molitor* larvae could degrade PUR foam by 35% in 17 days, assisted by gut microorganisms such as *Lactococcus* and *Pseudomonas* species [57,96]. Several studies have also demonstrated the potential of fungi to degrade PURs, such as *Cladosporium* sp. P7, which achieved a degradation rate of 94.5% of Impranil DLN-SD [52]. These findings suggest that microbial communities, both in soil and within insect digestive systems, play a crucial role in breaking down PUR materials [38,46,48,49,53,57,58,60,97,98,99,100,101]. Polymeric samples exhibited substantial degradation when exposed to microbial strains, as evidenced by weight reduction resulting from the adherence of microbial spores, which utilized polymeric substrate as a food and carbon source [102,103]. PUR samples treated with five bacterial and four fungal cultures showed varying rates of weight reduction. Weight reduction was initiated by the formation of a biofilm over a polymeric surface by bacterial and fungal cultures. An observable increase in the proliferation rate of the biofilm cells occurs with an increase during the incubation period [104,105,106], leading to partial degradation [107].

The most significant weight reductions were observed in samples treated with the coccus (C3) and *A. niger* species. In contrast to the cocci, other bacterial cultures showed little to no degradation. Among the coccus isolates, C2 exhibited the least percentage of weight reduction due to the slow formation of microcolonies, which subsequently affected the degradation rate. Prolonged incubation periods enhanced the likelihood and extent of degradation. The results of this current study align with or even surpass previous findings, such as the bacterial degradation of LDPE shopping bags, which achieved a 22.8% weight loss over 90 days of incubation [95]. In this current study, the LPDE samples treated with coccus (C3) achieved a 50% weight reduction, while polymer samples treated with *A. niger* showed a 40% weight reduction.

*A. clavatus* and *A. terreus* exhibited comparatively reduced weight loss compared to other strains. This limited degradative potential, reflected in the weight reduction rate, can be attributed to their inability to form a biofilm on the polymeric surface, thereby failing to trigger significant degradation. The hydrophobicity of the bacterial environment, in the absence of a carbon source, was favorable for microbial adhesion to the polymeric surface [107,108,109]. Polymeric samples treated with rod-shaped bacteria (B) exhibited the least weight reduction, amounting to only 10% of the initial weight. After isolating fungal and bacterial cultures from the dumpsite pedospheric region, they were identified based on macroscopic and microscopic characteristics. The results of the analysis confirmed the identification of the fungal strains *A. flavus*, *A. terreus*, *A. clavatus* and *A. niger* (Table 2), while the bacterial cultures were classified as rod-shaped bacteria, coccus and filamentous forms (Table 3).

The weight of the polymeric samples was compared before and after the addition of the fungal or bacterial cultures. Throughout the procedure, the polymer samples were left undisturbed to ensure that the fungal and bacterial cultures utilized the polymers as their sole carbon source to meet their nutritional requirements. This utilization indirectly reflected the deterioration of the polymeric structure, leading to its conversion. This assay demonstrated the total reduction in polymeric weight over the experimental period. The final weight of the polymer samples expressed a profound reduction, indicating the potential of fungal and bacterial biotechnological tools in degrading the selected polymeric waste materials (Table 4 and Table 5). Among fungal cultures, *A. niger* exhibited the highest weight reduction, while coccus bacterial cultures showed the most pronounced weight reduction in both polymeric samples. The weight reduction in LDPE samples treated with *A. niger* and coccus bacterial cultures was 40 and 50%, respectively. For PUR samples, the reduction was 55% with *A. niger* and 40% with the coccus bacterial culture.

**Figure 2:**
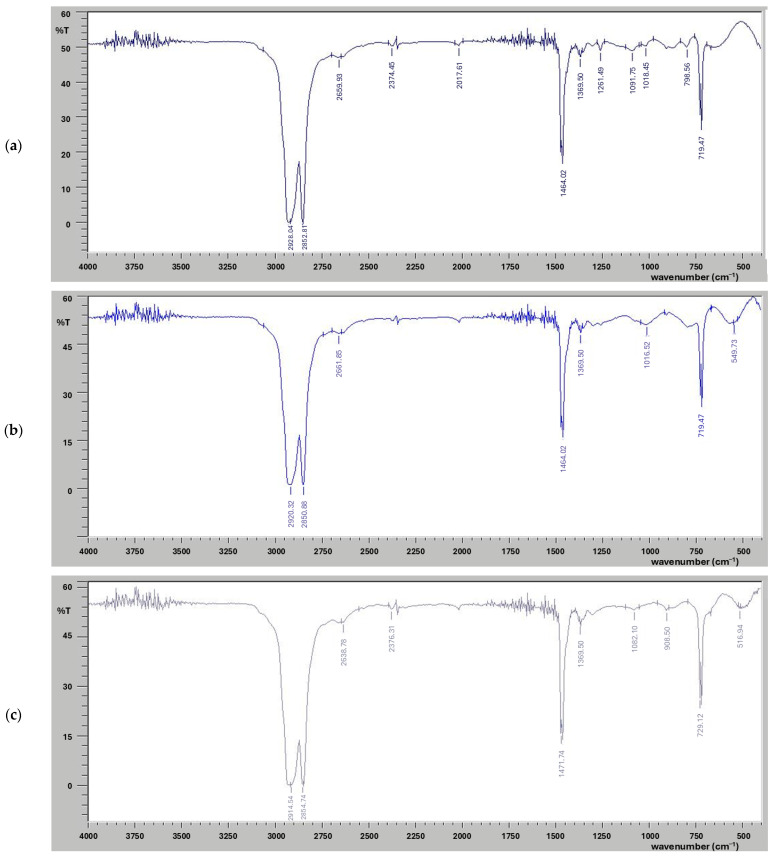
FT-IR spectra exhibiting the organic chemical profiles of the selected synthetic polymer LDPE, (**a**) control LDPE sample, (**b**) LDPE sample treated with fungal culture *Aspergillus niger* and (**c**) LDPE sample treated with bacterial strain coccus (C3).

FT-IR analysis was conducted to evaluate the breakdown products of the reference and pedospheric microbe-treated samples, as reflected by vibrational parameters shown in Figure 2. FT-IR spectra of the selected polymer LDPE and PUR exhibited a variety of peaks in response to microbial treatment. The controlled LDPE sample displayed numerous peaks, as shown in Figure 2a. Absorbance peaks formed at 2928 cm^−1^ and 2852 cm^−1^ indicated the presence of C-H bonds, signifying the presence of alkanes. An absorbance peak of variable strength at 1464 cm^−1^ showed the presence of a benzene ring, represented as C=C. Strong peaks were identified at 1091 cm^−1^ and 1369 cm^−1^, indicating the formation of stretching of C-O bonds. The peak at 2659 cm^−1^ is clearly indicative of the presence of alcoholic OH groups. Other peaks at 798 and 719 cm^−1^ depict the presence of =C-H bending bonds. The FT-IR spectra of the LDPE sample inoculated with *A. niger* revealed changes in the peaks corresponding to C-H bonds (alkanes), transitioning from 2928 cm^−1^ to 2920 cm^−1^ and from 2853 cm^−1^ to 2850 cm^−1^ (Figure 2b). The peak associated with C-O bonds steadily occurred at 1369 cm^−1^, and the peak corresponding to benzene rings also remained unaltered after the microbial treatment. FT-IR spectra of the LDPE sample incubated with coccus exhibited variability in the absorbance peaks (Figure 2c). The peak corresponding to C-H bonds at 2928 cm^−1^ shifted to 2914 cm^−1^, indicating a weakening of the bond. A significant increase in benzene ring peak from 1464 cm^−1^ to 1471 cm^−1^ was notable. Furthermore, there was a transition in the peak associated with the strong stretch of C-H bonds, which shifted from 1092 cm^−1^ to 1082 cm^−1^.

**Figure 3:**
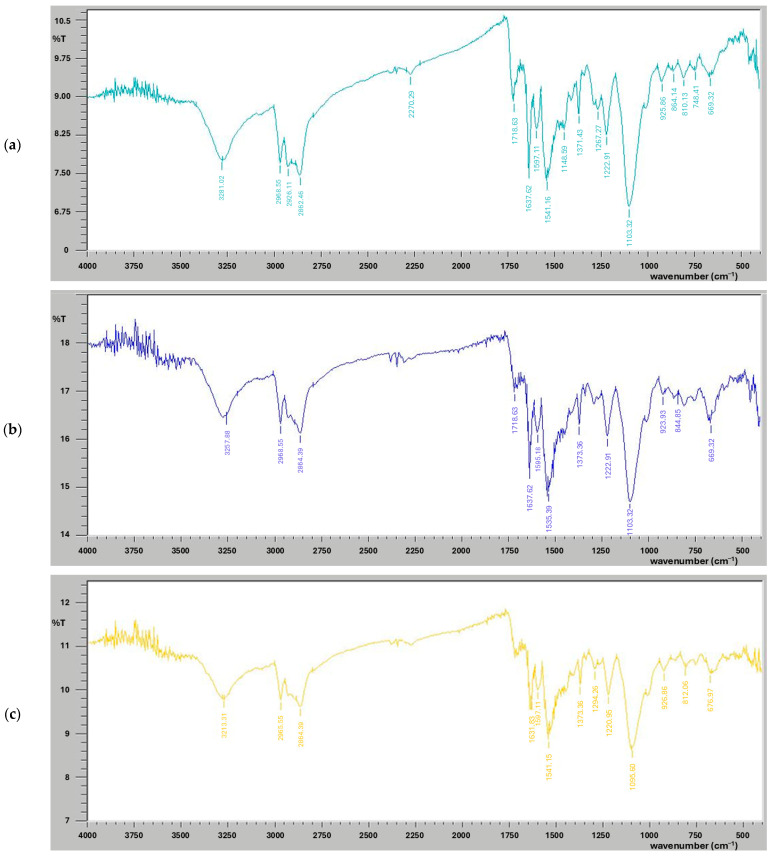
FT-IR spectra exhibiting the organic chemical profiles of the synthetic polymer PUR, (**a**) control PU sample, (**b**) PUR sample treated with fungal culture *Aspergillus niger* and (**c**) PUR sample treated with bacterial strain coccus (C3).

PUR polymeric samples were also analyzed using FT-IR to identify the functional groups present, as shown in Figure 3. The FT-IR spectra of reference PUR samples, which were not treated with any microbes, displayed various peaks (Figure 3a). An absorbance peak at 3281 cm^−1^ corresponded to the presence of a strong O-H bond. Peaks at 2968 cm^−1^, 2926 cm^−1^ and 2862 cm^−1^ indicated the presence of alkane C-H stretches with strong intensity. The FT-IR spectra of PUR samples treated with *A. niger* (Figure 3b) showed significant conversion, including the shift in the O-H bond peak from 3277 cm^−1^ to 3273 cm^−1^. While the peak for alkanes remained unaltered, a second peak stretched from 2862 cm^−1^ to 2864 cm^−1^. The alkene peak changed from 1637 cm^−1^ to 1631 cm^−1^, and the benzene peak transitioned from 1535 cm^−1^ to 1541 cm^−1^. A notable shift occurred in the absorbance peak of the strong O-H bond from 3281 cm^−1^ to 3257 cm^−1^. The aromatic C=C stretch absorbance peak shifted from 1541 cm^−1^ to 1535 cm^−1^. Microbial degradation of urethane bonds has been assumed to occur through ester bond hydrolysis [91]. The results of this current work regarding the bacterial decomposition of PUR (Figure 3c) align with previous studies on polymeric biodegradation using different microbial strains [89,91,107,110]. The absorbance peak at 2968 cm^−1^ remained unchanged in all bacterial samples after incubation, indicating no alterations in the C-H bonds and no evidence of cleavage. However, the decrease in the aromatic group C=C stretch values from 1541 cm^−1^ to 1533 cm^−1^, 1535 cm^−1^, 1518 cm^−1^ and 1527 cm^−1^ can be attributed to hydrolysis mediated by microbial esterase enzymes. This process excludes the likelihood of urease and protease enzymes performing other biochemical actions [110]. The disruption of PUR was confirmed by ester bond hydrolysis [91]. The observed stretching in the aromatic compound serves as an indicator of PUR degradation.

**Figure 4:**
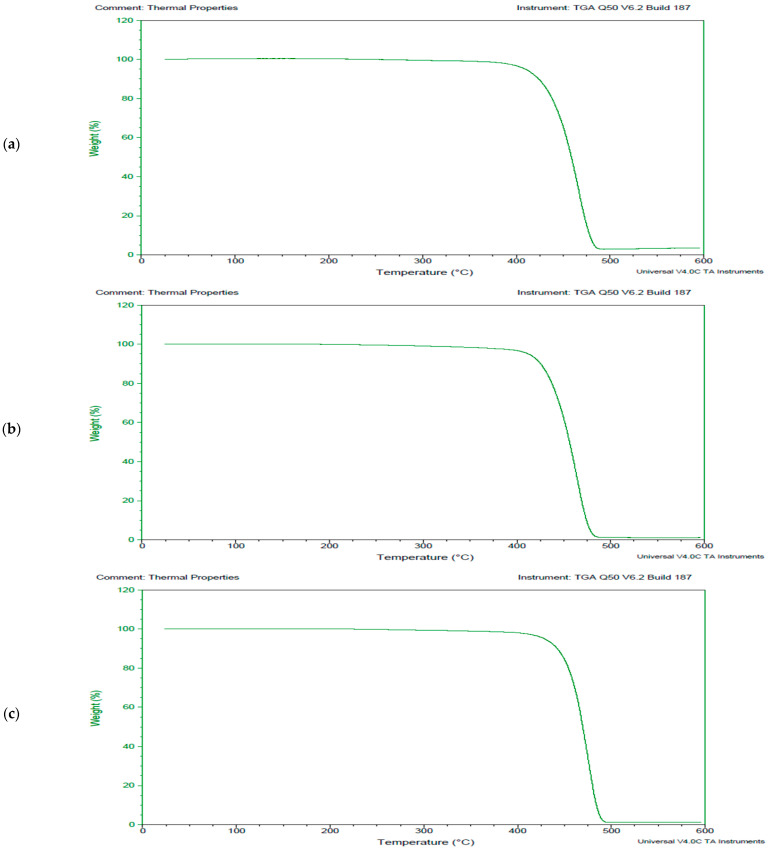
Thermograms exhibiting the breakdown pattern of the selected synthetic polymer LDPE, (**a**) thermogram exhibiting control LDPE sample, (**b**) thermogram exhibiting LDPE sample treated with fungal culture *Aspergillus niger* and (**c**) thermogram exhibiting LDPE sample treated with bacterial strain coccus (C3).

The LDPE polymer degraded by fungal and bacterial cultures was analyzed for thermal parameters using TGA. The biodegradation of LDPE via *A. niger*, shown in Figure 4, indicates that the process occured as single-step degradation. Initially, the weight of the LDPE polymer sample remained stable as the temperature increased to 420 °C, where a peak was observed due to sudden weight loss of the sample. This was followed by a consequential reduction in weight up to 95% at 480 °C, after which stability in weight loss was observed. A comparative analysis of the untreated reference sample and the *A. niger*-treated sample demonstrated the efficacy of the fungal biotechnological tool in degrading LDPE. In the untreated sample, the mass remained stable at 100% up to 430 °C, at which point 15% weight loss occurred. The *A. niger*-treated sample exhibited similar thermal behavior compared to the control LDPE sample, with only one peak observed at 425 °C. The degradation of the polymer began at 420 °C, and complete mass loss was observed at approximately 485 °C.

**Figure 5:**
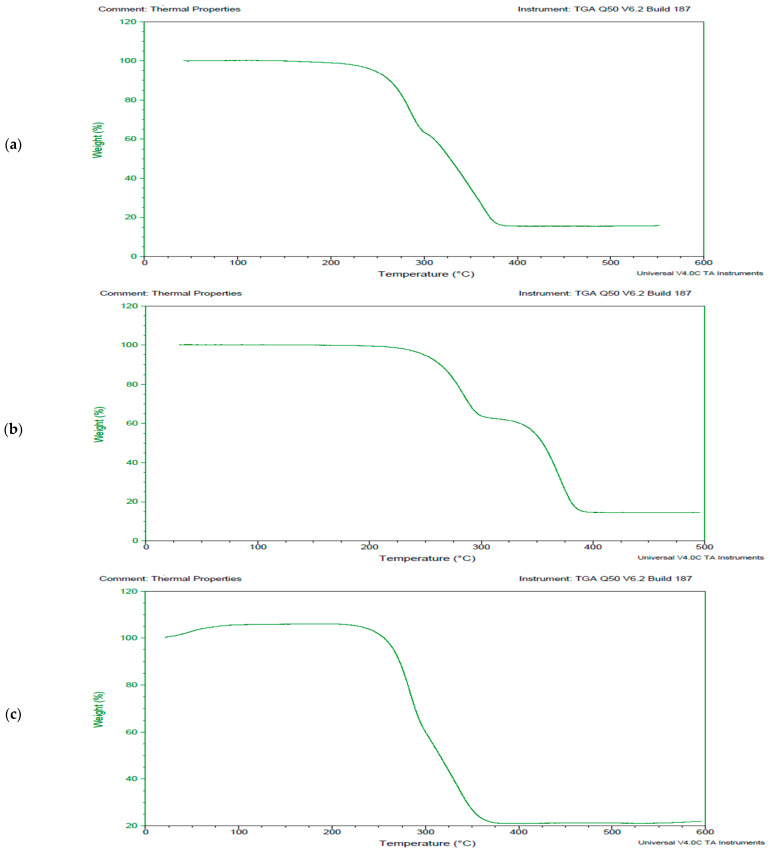
Thermograms exhibiting the breakdown pattern of the selected synthetic polymer PUR, (**a**) thermogram exhibiting control PU sample, (**b**) thermogram exhibiting PUR sample treated with fungal culture *Aspergillus niger* and (**c**) thermogram exhibiting PUR sample treated with bacterial strain coccus (C3).

TGA was performed to understand the thermal behavior of the PUR polymer in the context of microbial degradation (Figure 5). The TGA results indicated that the degradation of PUR occurred as a two-step process. Initially, the polymer remained stable as the temperature increased. However, when it reached 260 °C, degradation began, resulting in a 58% weight reduction by 290 °C. A second peak was observed at 290 °C, marking the onset of further mass loss, which continued until 375 °C. At this point, the total mass lost was about 85%. Beyond this temperature, the weight loss stabilized despite further temperature increases. The *A. niger*-treated PUR sample exhibited two distinct peaks. The first peak began at 250 °C and ended at 290 °C, corresponding to a weight loss of approximately 40%. The second peak started at 290 °C and ended at 380 °C, with a weight loss of about 90% (Figure 5b). The thermal behavior of the treated PUR polymer sample shifted from a two-stage to a one-stage degradation process. Degradation commenced at 40 °C, and the curve became linear up to 265 °C. Only one peak was observed at 265 °C. Notably, another peak present in the untreated PUR sample was absent in the treated sample. A maximum mass loss of 80% was observed at 375 °C.

**Figure 6:**
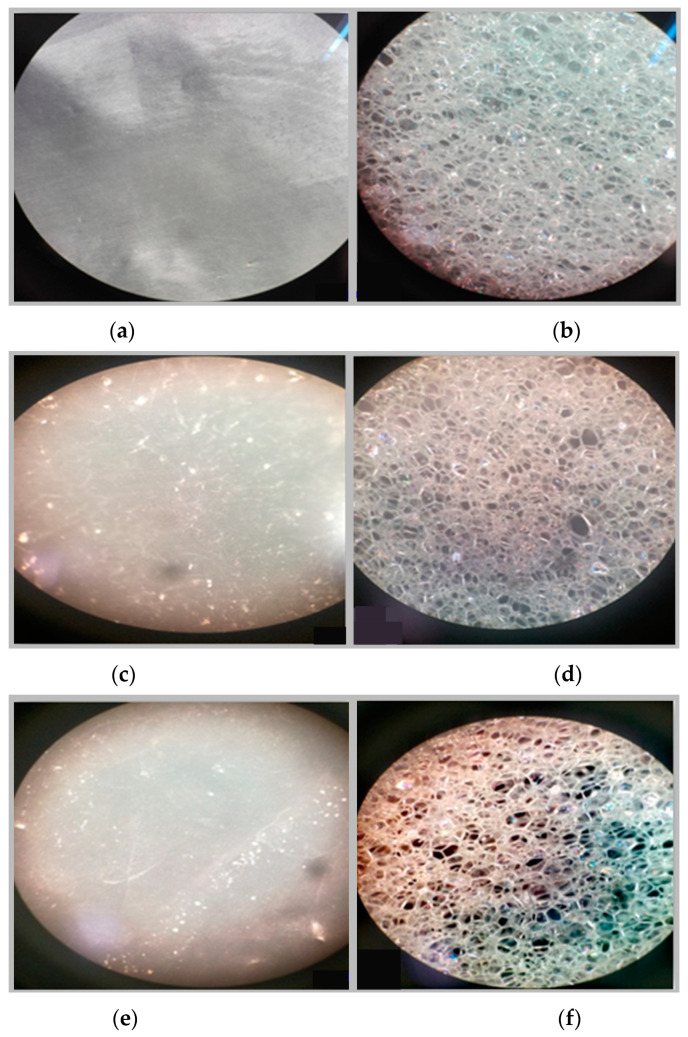
Stereomicroscopic images of the selected synthetic polymers, (**a**) untreated LDPE, (**b**) untreated PUR, (**c**,**d**) *Aspergillus niger*-driven biodegradation in LDPE and PUR, respectively, and (**e**,**f**) coccus (C3)-driven biodegradation in LDPE and PUR, respectively.

In addition to the vibrational and thermal analyses, microbially degraded LPDE and PUR samples were also analyzed by stereomicroscopy and SEM for morphological variations in the treated samples. The sterile LDPE and PUR degraded samples were examined under a microscope to obtain a detailed three-dimensional view (Figure 6). Bio-eroded surfaces were identified as the primary cause of weight reduction [95]. Microscopic images provided clear evidence of the successful deterioration of the polymeric substances following microbial treatment. The untreated LDPE sample exhibited a plain surface, whereas the untreated PUR sample (foam) displayed an irregular surface with roughness. Small, compactly bounded holes were also observed. After incubating the samples with fungal and bacterial cultures for four weeks, physical degradation was evident from stereomicroscopic images. The degradation manifested as pits, cracks and holes on the polymer surfaces, caused by the action of fungal and bacterial spores. The extent of degradation varied across microbial cultures, with some demonstrating significant deterioration. Initially, the controlled LDPE sample appeared very smooth, while the controlled PUR sample was compact. Post-incubation stereomicroscopic analysis revealed clear signs of degradation induced by bacterial cultures. For instance, the coccus-treated LDPE sample exhibited substantial surface changes, characterized by numerous small holes and dark spots, indicating surficial erosion.

**Figure 7:**
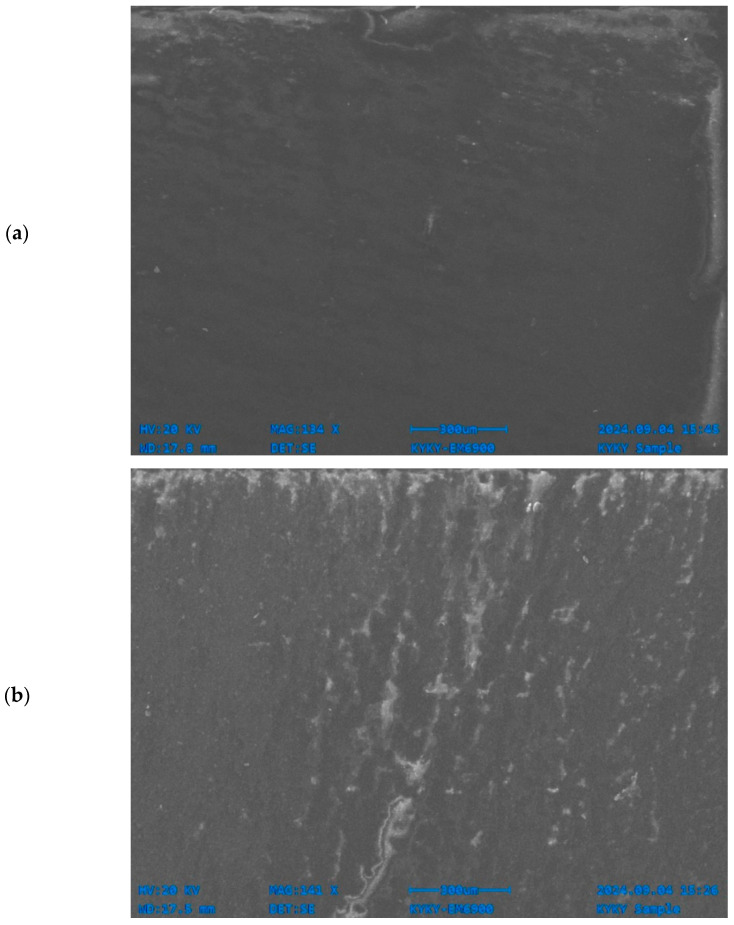
Scanning electron microscopic images of the degradation of LPDE by *Aspergillus niger* in liquid medium: (**a**) control and (**b**) *Aspergillus niger*-driven biodegradation in LDPE.

SEM analysis revealed prominent degradation on the surface of polymer films, indicating the effective biodegradative capacity of the fungal strains used. This effect was particularly observed within LDPE films (Figure 7). It is assumed that the fungi caused structural changes in the films by secreting enzymes that break down the material and utilizing polyethylene as a carbon source for their growth. Fungal cultures have been reported to metabolize polyethylene, leading to weight reduction in the film [111]. In our hands, the *A. niger* stain demonstrated a weight reduction of 45% in a liquid medium and 9% in soil. The lower degradation efficiency in soil is attributed to the more favorable growth conditions in the liquid medium. These findings also highlight the importance of environmental factors in influencing the biodegradation of polyethylene films.

## 4. Conclusions

Plastics and other polymeric products pose a significant threat, not only to humans and animals but also to the entire ecosphere. The persistence and resistance of polymers to various degradation mechanisms exacerbate their impact on an already contaminated global environment. Among the diverse approaches to address this issue, the use of microbial species as novel biotechnological tools for polymeric waste degradation emerges as a sustainable and cost-effective solution.

This research successfully utilized fungal and bacterial cultures isolated and identified from a local dumpsite, employing them as environmental remediators against LDPE and PUR polymers. Enhanced polymeric degradation driven by pedospheric microbes was achieved under controlled conditions, as reflected in FT-IR, TGA and stereomicroscopic analyses. Results showed that *A. niger* from fungal cultures and coccus among bacterial cultures contributed to the highest weight reduction in the polymeric samples. Weight reductions of 40% to 50% were observed for LDPE samples, and weight reductions of 40% to 55% were observed for PUR samples. Stereomicroscopic images clearly demonstrated surface morphological changes in the polymers before and after microbial treatment. The combined observations from stereomicroscopy, FT-IR spectral changes and TGA thermal degradation patterns strongly corroborate the occurrence of the biodegradation process. Notable, the FT-IR and TGA analyses also suggested potential alterations in the crystallinity of high-density polyethylene during microbial biodegradation, although further elucidation by SEM or atomic force microscopy is recommended. An additional important aspect that points toward further investigations related to the fungal strains isolated is that *Aspergillus* sp. are known for their ability to synthesize mycotoxins, such as aflatoxins in a strain-dependent manner, and are conditioned by environmental factors. As a part of the utility assessment of these strains to microbial decomposition of polymer waste materials, it must be confirmed that they do not produce aflatoxins. While this study primarily focused on the biodegradation potential of these microbes, the possible presence of aflatoxins was not assessed—a critical consideration for future research in order to ensure the safety and environmental sustainability of these strains in bioremediation. The promising results of this study highlight the practical viability of using pedospheric microbes as green and cost-effective solutions in polymeric waste remediation. Future research should explore optimizing these microbial strains to achieve higher degradation efficiencies. Additionally, the identification and evaluation of other pedospheric microbes for polymer waste disintegration may yield valuable insights and further advancements in this field.

## Figures and Tables

**Table 1:** Genus of bacterial cultures and their codes.

Bacteria	Colony Color	Code
Coccus	Orange	C1
Coccus	Light yellow	C2
Coccus	Pink	C3
Filamentous	Dark yellow	F
Rod shaped bacteria	White	B

**Table 2:** Microscopic and macro-based identification of fungal species.

Fungal Species	Microscopic Characteristic	Macro-Based Characteristic	Microscopic Image
*Aspergillus niger*	Spores appear to be dark brown or black in color.	The colony, dense and thick and black in color, grows within two or three days.	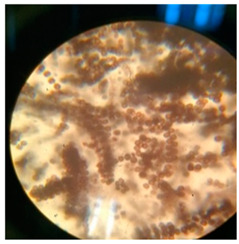
*Aspergillus flavus*	Spores appear to be yellowish green to dark green. These spores are connected to large fungal hyphae.	The colony, light green to yellowish green in color, with a smooth surface, grows within a week.	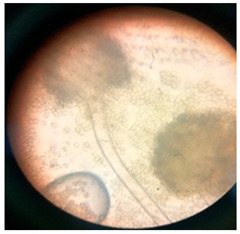
*Aspergillus clavatus*	Microscopic identification revealed the fungal body to be club shaped, having a spore color of medium brown to dark brown.	The colony, light green to olive green in color, grows within 2 days, and exudates are also visible on the surface.	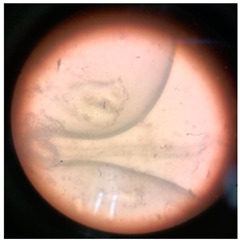
*Aspergillus terreus*	Identified by its short and round conidia attached to long hyphae, the color of these conidia is yellowish green to light green.	The colony, yellowish brown in color, grows very quickly within a few days.	* 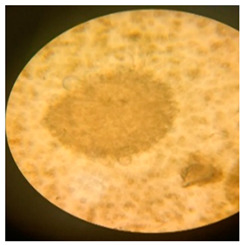 *

**Table 3:** Microscopic and macro-based identification of bacterial cultures.

Bacterial Colony Color	Stain Color	Shape	Characteristics	Microscopic Image
Light yellow	Purple	Round	Gram+, Coccus	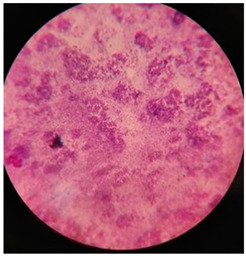
Orange	Purple	Round	Gram+, Coccus	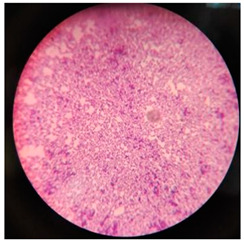
Pink	Purple	Round	Gram+, Coccus	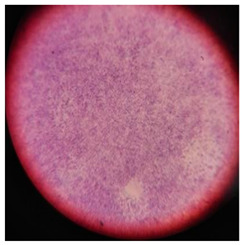
Dark yellow	Purple	Filamentous	Gram+, Filamentous	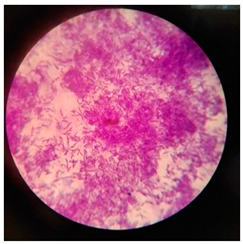
White	Pink	Rod	Gram−, rod-shaped bacteria	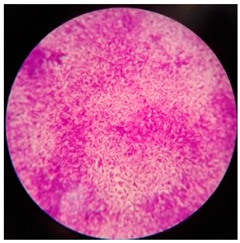

**Table 4:** Final weight of the selected synthetic polymers, low-density polyethylene (initial weight: 0.010 g) and polyurethane (initial weight: 0.011 g), upon treatment with different fungal species.

Polymer	Type of Fungal Culture	Final Weight of Samples (g)
Polyethylene	*Aspergillus flavus*	0.007
	*Aspergillus niger*	0.006
	*Aspergillus clavatus*	0.009
	*Aspergillus terreus*	0.008
Polyurethane	*Aspergillus flavus*	0.008
	*Aspergillus niger*	0.005
	*Aspergillus clavatus*	0.006
	*Aspergillus terreus*	0.009

**Table 5:** Final weight of the selected synthetic polymers, low-density polyethylene and polyurethane, upon treatment with different bacterial strains.

Polymer	Bacterial Species	Colony Color	Final Weight of Samples (g)
Polyethylene	*Coccus* sp.	Orange	0.006
	*Coccus* sp.	Pink	0.005
	*Coccus* sp.	Light yellow	0.009
	Rod-shaped bacteria sp.	White	0.009
	*Filamentous* sp.	Dark yellow	0.008
Polyurethane	*Coccus* sp.	Orange	0.008
	*Coccus* sp.	Pink	0.007
	*Coccus* sp.	Light yellow	0.010
	Rod-shaped bacteria sp.	White	0.009
	*Filamentous* sp.	Dark yellow	0.010

## Data Availability

The data presented in this study are contained within the manuscript.
